# Molecular catalysis at polarized interfaces created by ferroelectric BaTiO_3_ †
†Electronic supplementary information (ESI) available. See DOI: 10.1039/c6sc05032h



**DOI:** 10.1039/c6sc05032h

**Published:** 2017-02-06

**Authors:** Eugene S. Beh, Sergey A. Basun, Xiaofeng Feng, Ighodalo U. Idehenre, Dean R. Evans, Matthew W. Kanan

**Affiliations:** a Department of Chemistry , Stanford University , 337 Campus Drive , Stanford , California 94305 , USA . Email: mkanan@stanford.edu; b Air Force Research Laboratory , Materials and Manufacturing Directorate , Wright-Patterson Air Force Base , Ohio 45433 , USA; c Azimuth Corporation , 4134 Linden Avenue, Suite 300 , Dayton , Ohio 45432 , USA; d University of Dayton , Department of ECE and Electro-Optics Program , Dayton , Ohio 45469 , USA

## Abstract

The local environment at polarized solid–liquid interfaces provides a unique medium for chemical reactions that could be exploited to control the selectivity of non-faradaic reactions. Polarized interfaces are commonly prepared by applying a voltage to an electrode in an electrolyte solution, but it is challenging to achieve high surface charge densities while suppressing faradaic reactions. Ferroelectric materials have permanent surface charge densities that arise from the dipole moments of ferroelectric domains and can be used to create polarized solid–liquid interfaces without applying a voltage. We studied the effects of ferroelectric oxides on the selectivity of a Rh porphyrin-catalyzed carbene rearrangement. The addition of ferroelectric BaTiO_3_ nanoparticles to the reaction solution changed the product ratio in the same direction and by a similar magnitude as performing the reaction at an electrode–electrolyte interface polarized by a voltage. The results demonstrate that colloidal suspensions of BaTiO_3_ nanoparticles act as a dispersible polarized interface that can influence the selectivity of non-faradaic reactions.

## Abstract 2

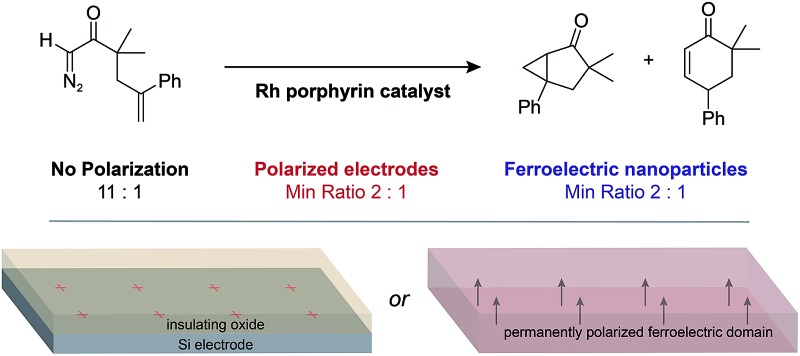
Colloidal suspensions of ferroelectric BaTiO_3_ nanoparticles act as a dispersible polarized interface that can influence the selectivity of non-faradaic reactions.

## Introduction

The double layer region at a polarized solid–liquid interface has electrostatic and chemical properties that differ significantly from a bulk solution.^1,2^ These properties affect the rates of electrochemical and photocatalytic reactions, which entail charge transfer across the interface.^3–6^ We previously investigated the effects of a polarized electrode–electrolyte interface on the selectivity of catalytic non-faradaic reactions.^7,8^ These experiments employed a custom reaction vessel—the “parallel plate cell”—whose walls consist of Si electrodes coated with thin layers of an insulating oxide. Polarization of the oxide–electrolyte interface by applying a voltage across the Si electrodes was found to change the selectivity of reactions confined to the interface, including an epoxide rearrangement catalyzed by Al_2_O_3_ and an intramolecular carbene rearrangement catalysed by Rh porphyrins. The selectivity changes were dependent on the charge density at the oxide–electrolyte interface, which was determined from the applied voltage and the interfacial capacitance.

**Fig. 1:**
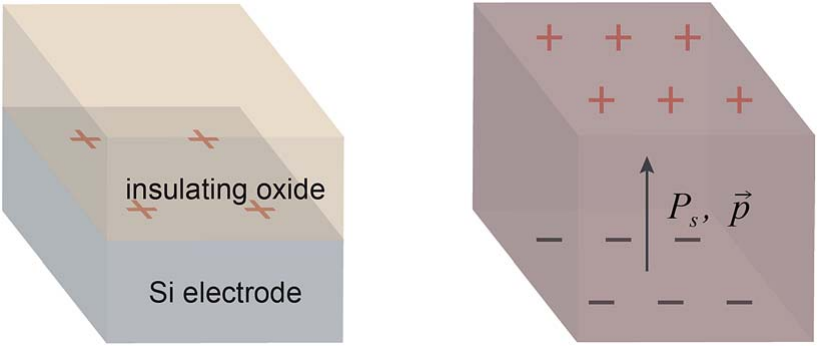
Depiction of the surface of a charged electrode coated with an insulating oxide (left) and a permanently polarized ferroelectric domain (right). The arrow denotes the direction of polarization of the domain.

The study of non-faradaic reactions at voltage-polarized interfaces has limitations. The need to block faradaic processes necessitates fabricating electrodes with thin insulating layers and dielectric breakdown of these layers limits the maximum interfacial charge density that can be achieved. To address these issues, we sought to replace an electrode polarized by a voltage with a material that permanently has a large surface charge density. Inorganic ferroelectrics are crystalline materials composed of domains that have permanent dipole moments below a characteristic Curie temperature.^9^ The surfaces of ferroelectric particles have regions of high charge density where the dipole moment of the domain at the surface has a normal component, leading to the formation of double layers.^10^ Previous studies of chemical reactivity at ferroelectric surfaces have utilized the polarization to localize and enhance photoredox reactions.^11–14^ We hypothesized that ferroelectric materials would provide polarized solid–liquid interfaces that affect the selectivity of non-faradaic reactions in the same manner as an electrode–electrolyte interface polarized by a voltage (Fig. 1).

Barium titanate (BaTiO_3_) is a readily accessible ferroelectric metal oxide with a Curie temperature of 120 °C.^15^ The polarization of domains in BaTiO_3_ arises from a small displacement of the Ti^4+^ cation from the centre of the tetragonal unit cell. The polarization of BaTiO_3_ is stable at room temperature.^16–18^

The charge density on a BaTiO_3_ surface that is normal to the dipole moment of a polarized domain can be estimated from previous measurements of the spontaneous polarization (*P*_s_) of BaTiO_3_ nanoparticles. The *P*_s_ of a BaTiO_3_ nanoparticle composed of a single ferroelectric domain corresponds to the charge densities on the opposing surfaces of the domain that are normal to the dipole moment (Fig. 1). Ball milling of bulk BaTiO_3_ powder produces nanoparticles that are composed of a small number of ferroelectric domains. We have measured *P*_s_ values of 20–120 μC cm^–2^ for ball milled BaTiO_3_ nanoparticles by performing triangular wave ac voltammetry on nanoparticle suspensions.^19,20^ (These values for *P*_s_ assume that each nanoparticle is a cube composed of a single ferroelectric domain; particle aggregation will lower the observed *P*_s_).^21,22^ Notably, surface charge densities of 20–120 μC cm^–2^ are considerably larger than what is accessible in the parallel plate cell at voltages below the dielectric breakdown limit (at most ∼14 μC cm^–2^).^8^ Larger BaTiO_3_ particles that have not been subjected to ball milling have little or no measurable *P*_s_ because they are composed of many ferroelectric domains that are randomly oriented and therefore cancel each other out. Nevertheless, the average magnitude of surface charge density on large BaTiO_3_ particles is expected to be similar to BaTiO_3_ nanoparticles because the charge density at any point on the surface is dominated by the orientation of the closest domain.

## Results and discussion

**Scheme 1:**
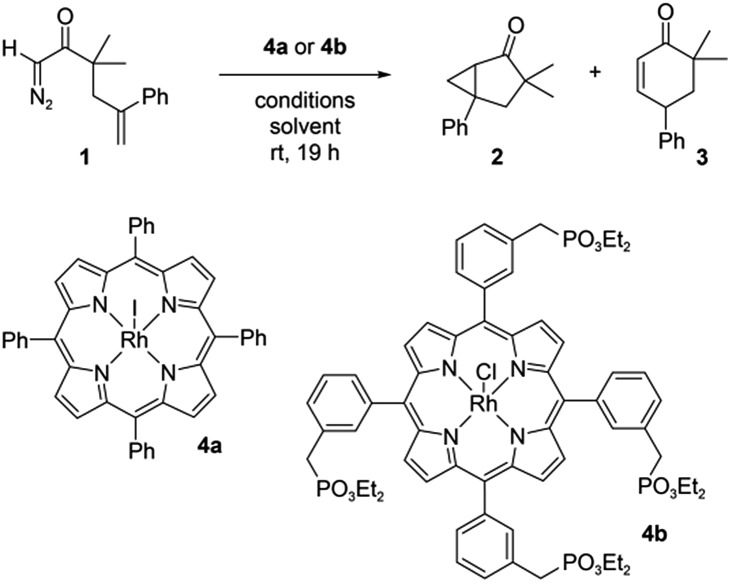
The reaction of diazo compound **1** catalyzed by Rh porphyrins **4a** or **4b** produces products **2** and **3**.

A Rh porphyrin-catalyzed intramolecular carbene reaction was used as a model reaction. Diazoketone **1** reacts at ambient temperature with Rh porphyrin catalysts to form a mixture of products **2** and **3** (Scheme 1). In the absence of a polarized interface, the reaction favours **2** over **3** by an approximately 10 : 1 ratio. We previously studied this same reaction in the parallel plate cell using an applied voltage to create polarized electrode–electrolyte interfaces.^8^ Rh porphyrin catalysts were localized to these interfaces using either covalent attachment or spontaneous physisorption. With Al_2_O_3_-coated electrodes or alkylphosphonate-coated electrodes, the **2** : **3** ratio decreased as the magnitude of the voltage was increased, reaching 2 : 1 at the maximum voltage that could be applied before dielectric breakdown. We attributed this change to an effect of the local solvation and electrostatic environment at the polarized interface on the competing activation barriers leading to **2** and **3**.

To see whether the effect of an interface polarized by a voltage could be recapitulated by a dispersed, permanently polarized interface, we compared the selectivity of the Rh porphyrin-catalyzed reaction of **1** to products **2** and **3** in the presence or absence of various ferroelectric (BaTiO_3_, PbTiO_3_, and LiNbO_3_) and non-ferroelectric (CaTiO_3_, SrTiO_3_, and TiO_2_) nanoparticles. The nanoparticle samples were produced as colloidal suspensions by ball milling the corresponding bulk powders in the presence of heptane and oleic acid.^19^ Transmission electron microscopy (TEM) analysis indicated average particle sizes in the range of 7–10 nm for all the milled materials except for LiNbO_3_ (50 nm; Fig. S1–S12†). The average *P*_s_ for the nanoparticles was calculated from the ac displacement current of nanoparticle suspensions subjected to a periodic ac electric field within a narrow cell.^20^ A *P*_s_ of 20 μC cm^–2^ was measured for the BaTiO_3_ nanoparticles, indicating substantial alignment of the ferroelectric domains. As expected for non-ferroelectric materials, the *P*_s_ values calculated from the ac displacement measurements for CaTiO_3_, SrTiO_3_, and TiO_2_ were all at least two orders of magnitude smaller than BaTiO_3_ (Fig. S13†).

Reactions were performed by diluting freshly ball milled 20 : 1 : 1 w/w/w heptane/oleic acid/nanoparticle suspensions to the appropriate nanoparticle concentration with a solution of Rh porphyrin catalyst **4a** in CH_2_Cl_2_, followed by a solution of **1** in CH_2_Cl_2_. The reactions were allowed to proceed for 19 h and then analysed using NMR and HPLC. **2** and **3** were the only major products observed (see ESI main text and Fig. S14†).

**Fig. 2:**
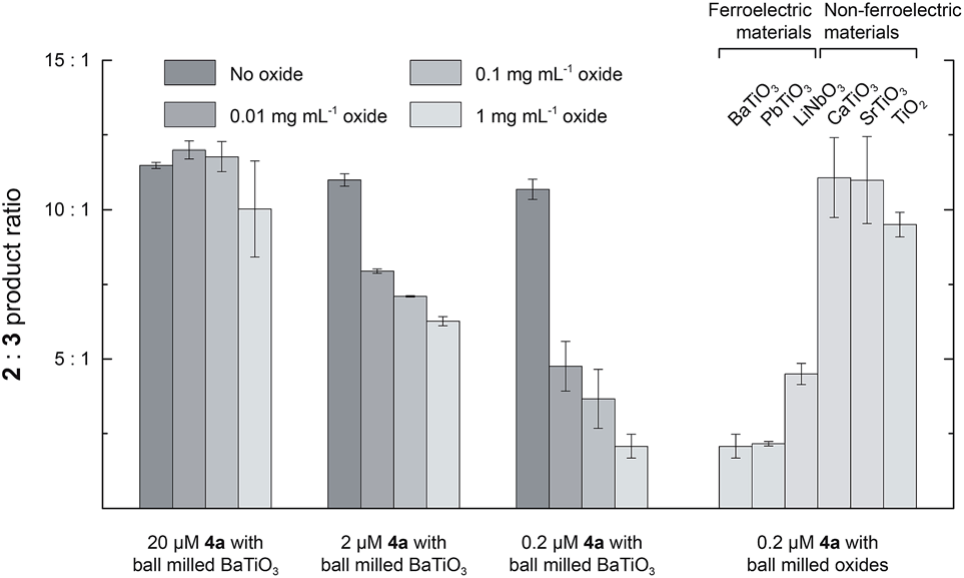
The dependence of product ratio **2** : **3** on the catalyst **4a** concentration and ball milled oxide concentration. All reactions were conducted with 2 mM of **1** in CH_2_Cl_2_ at room temperature for 19 h (0.2 mM of **1** when catalyst concentration is 0.2 μM). Data are averaged over two or three separate experiments; error bars represent the standard deviation in the observed product ratio.

Fig. 2 shows the **2** : **3** product ratio for reactions performed with different concentrations of BaTiO_3_ nanoparticles, **1**, and **4a** in CH_2_Cl_2_. In the absence of nanoparticles, the **2** : **3** ratio was ∼11 : 1 in all cases. The largest changes were observed with 0.2 mM **1** and 0.2 μM **4a**, to which the addition of increasing concentrations of BaTiO_3_ nanoparticles up to 1 mg mL^–1^ resulted in a 5.1-fold decrease of the product ratio from 10.7 : 1.0 to a minimum of 2.1 : 1.0. These changes are in the same direction as what was observed with externally polarized electrode–electrolyte interfaces in the parallel plate cell.^8^ Smaller changes were observed with higher ratios of catalyst to nanoparticles, most likely because of incomplete localization of the catalyst to the ferroelectric–solution interface (see below).

Under the same conditions with 1 mg mL^–1^ of nanoparticles, similar changes in the product ratio were also seen with other ferroelectric materials like PbTiO_3_ (2.1 : 1.0) and LiNbO_3_ (4.5 : 1.0). In contrast, very little effect on the product ratio was seen with chemically similar non-ferroelectric nanoparticles such as CaTiO_3_ (11.1 : 1.0), SrTiO_3_ (11.0 : 1.0), or TiO_2_ (9.5 : 1.0).

Control experiments in which **1** was combined with BaTiO_3_ nanoparticles in the absence of catalyst **4a** showed no conversion, which shows that the BaTiO_3_ is not itself a catalyst for the diazo decomposition. In addition, reactions performed in the presence of heptane and oleic acid without any nanoparticles gave a **2** : **3** ratio of 11.3 : 1.0, indicating that these additives themselves do not impact the product ratio.

The ability of the polarized BaTiO_3_–solution interface to affect the selectivity of **4a** requires that a substantial portion of the catalyst partitions at or near the BaTiO_3_ surface during the reaction. The concentration dependence of the BaTiO_3_ effect in the results above is consistent with an interface-dependent effect: as the ratio of BaTiO_3_ surface area to **4a** is increased, a larger change in product ratio is observed. Notably, the surface coverage of long aliphatic capping ligands at colloidal nanoparticles is much lower than one monolayer,^23^ which may be critical to allow the reactant and catalyst molecules to localize near the surface.

To further test this model, a reaction was performed in the presence of an excess of un-metalated 5,10,15,20-tetraphenylporphyrin (TPP), which could compete with **4a** for localizing to the surface. When 100 μM TPP was added to a reaction with 2 mM **1**, 2 μM **4a** and 1 mg mL^–1^ of BaTiO_3_ nanoparticles, the product ratio was 10.6 : 1.0 compared to 6.3 : 1.0 when TPP was omitted and 11.0 : 1.0 when both TPP and BaTiO_3_ were absent. This result shows that the BaTiO_3_ effect is lost in the presence of a species that can block the surface localization of the catalyst. Furthermore, when 2 μM **4a** was first stirred with 1 mg mL^–1^ of BaTiO_3_ nanoparticles for 24 h before the introduction of 100 μM TPP and 2 mM **1**, the ratio was 10.2 : 1.0 after a further 19 h of reaction time. This experiment demonstrates that simply exposing the catalyst to BaTiO_3_ nanoparticles does not result in an irreversible change to a species with different selectivity. Notably, if the anion of **4a** is replaced by chloride, a smaller change in product ratio from 8.1 : 1.0 (no BaTiO_3_) to 5.1 : 1.0 (1 mg mL^–1^ BaTiO_3_) is obtained, which may reflect reduced localization of the Cl-bound Rh porphyrin at the BaTiO_3_ surface.

**Fig. 3:**
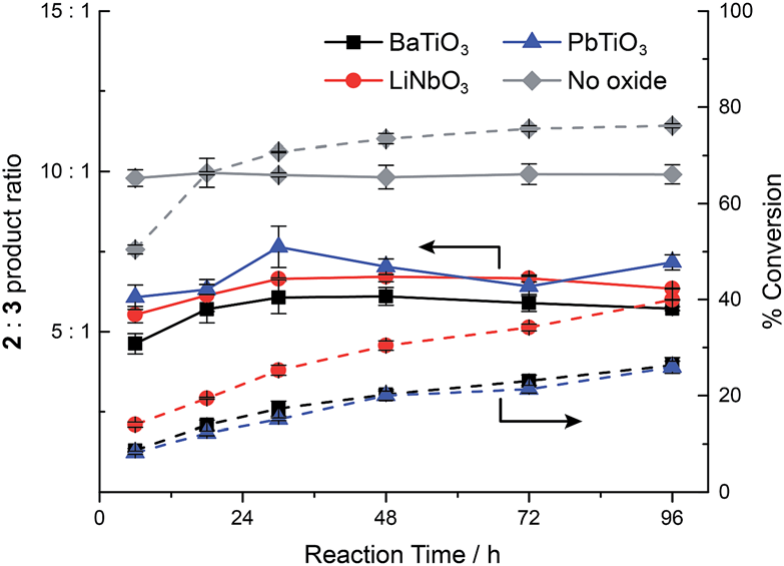
Evolution of the conversion (right axis, dashed lines) and product ratio **2** : **3** (left axis, solid lines) with time in the presence or absence of various ball milled ferroelectric oxides. Aliquots were taken from reactions that were run with 2 mM **1** and 2 μM **4a** in CH_2_Cl_2_ at room temperature. Ball milled oxide, if present, is used at a concentration of 1 mg mL^–1^.

In order to understand the timescale on which ferroelectric nanoparticles affect the catalyst, the reaction kinetics were studied in the presence or absence of the various ball milled ferroelectric oxides (Fig. 3). Solutions of 2 mM **1**, 2 μM **4a**, and 1 mg mL^–1^ of ball milled oxide (BaTiO_3_, PbTiO_3_, or LiNbO_3_) in approximately 10 mL of CH_2_Cl_2_ were prepared, as well as an identical reaction with no oxide. Ball milled oxides were used within 60 minutes after ball milling had concluded. Over the course of 96 h, aliquots from each of the reaction mixtures were quenched with acetonitrile and immediately analysed by HPLC to determine the conversion and ratio of products **2** and **3**. In each case, the product ratio stayed roughly constant throughout the course of 96 h, which suggests that catalyst molecules localize to the polarized ferroelectric surface within, at most, several hours. The slight increase in product ratio after the 6 h mark is attributed to some aggregation of the ball milled nanoparticles over the course of several hours, which resulted in a lowered surface area that was accessible to the catalyst.

**Fig. 4:**
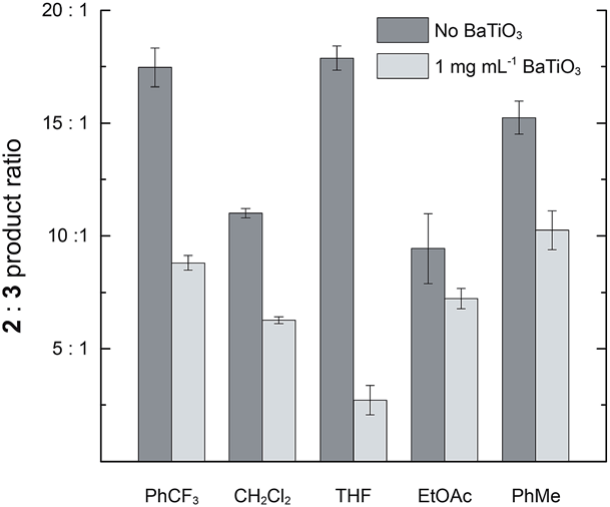
The effect of adding 1 mg mL^–1^ ball milled BaTiO_3_ nanoparticles on the product ratio **2** : **3** in a variety of solvents. Reactions were run with 2 mM **1** and 2 μM **4a** at room temperature for 19 h.

The effect of BaTiO_3_ nanoparticles was also examined in solvents other than CH_2_Cl_2_. Reactions were performed with 2 mM **1** and 2 μM **4a** in PhCF_3_, THF, EtOAc, and PhCH_3_. In the absence of BaTiO_3_ nanoparticles, the **2** : **3** ratio ranged from 9.4 : 1.0 to 17.9 : 1.0, demonstrating that the strong preference for cyclopropanation is maintained across diverse solvents. The addition of 1 mg mL^–1^ BaTiO_3_ nanoparticles caused a reduction in the **2** : **3** ratio in all cases, with a substantial variation in the magnitude of the effect (Fig. 4). The largest effect was a 6.6-fold reduction from 17.9 : 1.0 to 2.7 : 1.0 observed in THF. Solvents more polar than PhCF_3_ could not be used because they caused immediate flocculation of the nanoparticles.

**Fig. 5:**
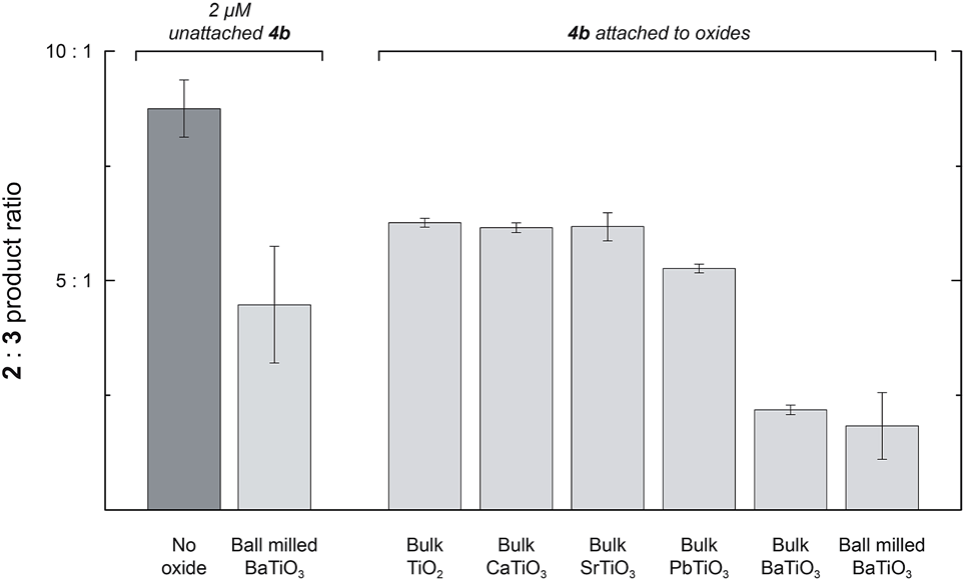
The product ratio **2** : **3** with catalyst **4b** unattached (2 μM, left two columns) or attached to various titanium-containing oxides (right columns). All reactions were conducted with 2 mM of **1** and 1 mg mL^–1^ of oxides (where present) in CH_2_Cl_2_ at room temperature. Reaction times were 19 h for all experiments, but 96 h for CaTiO_3_, SrTiO_3_, and PbTiO_3_ in order to obtain sufficient conversion for analysis.

Unmilled BaTiO_3_ particles have no measurable *P*_s_ because they are composed of randomly oriented domains (see above), but they still have regions of high surface charge densities. Adding 1 mg mL^–1^ of unmilled BaTiO_3_ particles to a reaction with 2 mM **1** and 2 μM **4a** caused only a 1.2-fold reduction in the **2** : **3** ratio, but the surface area of these relatively large particles (∼900 nm) is too low for effective physisorption of **4a** in solution. To test for an interfacial polarization effect on selectivity with unmilled BaTiO_3_, we prepared Rh porphyrin **4b** with four phosphonate groups to enable covalent surface attachment. The results of reactions performed with **4b** are summarized in Fig. 5. When used as a homogeneous catalyst, 2 μM **4b** reacted with 2 mM **1** in CH_2_Cl_2_ to give a **2** : **3** product ratio of 8.8 : 1.0. As with catalyst **4a**, the introduction of 1 mg mL^–1^ ball milled BaTiO_3_ resulted in a 2.0-fold decrease of the product ratio to 4.5 : 1.0. Next, **4b** was attached to various ferroelectric and non-ferroelectric oxides studied. Reactions performed with 2 mM **1** and 1 mg mL^–1^ unmilled BaTiO_3_ functionalized with **4b** gave a **2** : **3** product ratio of 2.2 : 1.0, corresponding to a 4.0-fold reduction. The **2** : **3** ratio was further reduced to 1.8 : 1.0 if the BaTiO_3_ functionalized with **4b** was first subjected to ball milling and then used at the same oxide concentration.

In contrast, when **4b** was attached to the non-ferroelectric oxides TiO_2_, CaTiO_3_, and SrTiO_3_, a small (1.4-fold) effect on the reaction to approximately 6.3 : 1 was observed. Measurements of zeta potentials for TiO_2_ particles in hexane in the presence of surfactant indicate surface charge densities on the order of –0.1 μC cm^–2^, which is much lower than the surface charge density for ferroelectric nanoparticles.^24^ The small change in selectivity observed upon attachment of **4b** to nonferroelectric nanoparticles may arise from the interaction of the catalyst with the oxide surface or a subtle conformational biasing for attached catalysts.^25,26^ When **4b** was attached to ferroelectric PbTiO_3_, the ratio was 5.3 : 1.0, which was only slightly lower than the other non-ferroelectric oxides. This result may arise from decreased robustness of the phosphonate linkage to the PbTiO_3_ surface and increased leaching of catalyst molecules into the bulk solution. Nevertheless, attachment to ferroelectric oxides, especially BaTiO_3_, effected a substantially larger change in selectivity than attachment to other non-ferroelectric oxides.

The results above demonstrate that the polarized interface between a ferroelectric oxide and an organic solvent can change the selectivity of catalytic reactions in the same way as an electrically polarized electrode–electrolyte interface. By attaching the catalyst to BaTiO_3_ surfaces or adding BaTiO_3_ nanoparticles to the reaction solution, the **2** : **3** ratio is reduced by up to a factor of 4.9. Similar effects are seen with ball milled ferroelectric PbTiO_3_ and LiNbO_3_; chemically similar but non-ferroelectric CaTiO_3_, SrTiO_3_, or TiO_2_ nanoparticles had essentially no effect, which indicates that the selectivity change requires the permanently polarized domains in BaTiO_3_.

## Conclusions

By using ball milled ferroelectric nanoparticles such as BaTiO_3_, a polarized interface can now be introduced to a reaction medium without the need for physical electrodes. The use of ferroelectric nanoparticles provides an accessible way to investigate and exploit the effects of interfacial polarization on catalysis.
