# Supplementary material for: Molecular catalysis at polarized interfaces created by ferroelectric BaTiO3
Source: Chem Sci. 2017 Feb 6;8(4):2790–4. doi: 10.1039/c6sc05032h (PMC5426436; doi:10.1039/c6sc05032h)
Supplement: Supplementary file 1 [file SC-008-C6SC05032H-s001.pdf]

Electronic Supplementary Material (ESI) for Chemical Science.
This journal is © The Royal Society of Chemistry 2017

Electronic Supplementary Information

Molecular Catalysis at Polarized Interfaces Created by Ferroelectric $BaTiO_3$

Eugene S. Beh,[‡] Sergey A. Basun,[§,‖] Xiaofeng Feng,[¶] Ighodalo U. Idehenre,[§,=] Dean R. Evans[§] and Matthew W. Kanan[*]

* To whom correspondence should be addressed. Department of Chemistry, Stanford University, 337 Campus Drive, Stanford, California 94305, USA. E-mail: mkanan@stanford.edu

‡ Current address: Department of Chemistry and Chemical Biology, Harvard University, 12 Oxford Street, Naito 118, Cambridge, Massachusetts 02138, USA

§ Air Force Research Laboratory, Materials and Manufacturing Directorate, Wright-Patterson Air Force Base, Ohio 45433, USA

‖ Azimuth Corporation, 4134 Linden Avenue, Suite 300, Dayton, Ohio 45432, USA

¶ Department of Physics, University of Central Florida, Orlando, Florida 32816, USA

= University of Dayton, Department of ECE and Electro-Optics Program, Dayton, Ohio 45469, USA

Index		*Page*
Experimental methods and additional experimental data		S2–S5
**Figure S1.**	Representative TEM image of bulk $BaTiO_3$ particles	S6
**Figure S2.**	Representative TEM image of ball milled $BaTiO_3$ nanoparticles	S6
**Figure S3.**	Representative TEM image of bulk $PbTiO_3$ particles	S6
**Figure S4.**	Representative TEM image of ball milled $PbTiO_3$ particles	S6
**Figure S5.**	Representative TEM image of unmilled $LiNbO_3$ particles	S7
**Figure S6.**	Representative TEM image of ball milled $LiNbO_3$ particles	S7
**Figure S7.**	Representative TEM image of unmilled $CaTiO_3$ particles	S7
**Figure S8.**	Representative TEM image of ball milled $CaTiO_3$ particles	S7
**Figure S9.**	Representative TEM image of unmilled $SrTiO_3$ particles	S8
**Figure S10.**	Representative TEM image of ball milled $SrTiO_3$ particles	S8
**Figure S11.**	Representative TEM image of unmilled TiO_2 particles	S8
**Figure S12.**	Representative TEM image of ball milled TiO_2 particles	S8
**Figure S13.**	Measurements of displacement current for selected ball milled oxides	S9
**Figure S14.**	Sample NMR spectrum showing **1**, **2** and **3**	S10
	Experimental data for **Figure 2**	S11
	Experimental data for **Figure 3**	S12
	Experimental data for **Figure 4**	S14
	Experimental data for **Figure 5**	S15
References		S16

Experimental Methods

General.

Unless otherwise specified, all solvents were obtained from Fisher Chemical (ACS grade) and used as received. $PhCF_3$ was purchased from Sigma-Aldrich and used as received.

Preparation of reagents and catalysts.

1, **4a** and **4b** were synthesized and purified according to the literature.[1]

Ball milling. Bulk $BaTiO_3$ (2 µm), $LiNbO_3$ (size not specified), TiO_2 (anatase phase, size not specified) and $SrTiO_3$ (<5 µm) powders were purchased from Sigma-Aldrich. Bulk $PbTiO_3$ (325 mesh, 44 µm) and $CaTiO_3$ powder (size not specified) was purchased from Strem Chemicals. (See below for unmilled particle sizes as determined by TEM.) Ball milling was performed with a Fritsch Pulverisette 7 Planetary Micro Mill Classic with 12 mL zirconium oxide grinding bowls. In a grinding bowl, 20 g of 2 mm diameter zirconium oxide grinding balls, 1.6000 g of heptane, and 80.0 mg of oleic acid were added, followed by either 80.0 mg of $BaTiO_3$, 80.0 mg of $PbTiO_3$, 80.0 mg of $LiNbO_3$, 80.0 mg of $SrTiO_3$, 40.0 mg of $CaTiO_3$, or 40.0 mg of TiO_2. The bowl was sealed and spun in the ball mill for 16 cycles of 60 minutes at 500 rpm, then 30 minutes of rest. The direction of spinning was reversed after each cycle. The total time for the procedure was 24 hours.

Once ball milling was complete, the grinding bowl was opened and the nanoparticle suspension pipetted out into a glass vial for use in reactions. In order to remove residual oxide, grinding bowls and balls were thoroughly rinsed with hexane and sonicated several times in methanol, then briefly soaked in concentrated nitric acid for about 5 minutes and thoroughly washed with deionized water before being used again.

TEM analysis of bulk and ball milled oxides. TEM analysis was performed using an FEI Tecnai G2 F20 X-TWIN Transmission Electron Microscope operating at 200 kV. Samples were prepared by drop-casting dilute suspensions of the various oxides in methanol (for bulk powders) or heptane (for ball milled nanoparticles) onto copper TEM grids. Particle and nanoparticle sizes were estimated by measuring 100 different particles, from which the average size and standard deviation were calculated. Representative TEM images of the samples are shown in **Figures S1–S14**. Notably, bulk and ball milled $LiNbO_3$ particles had a flat, plate-like morphology, unlike the more granular appearance of the other oxides. The entry for $LiNbO_3$ reflects the particle width and not the thickness, which could not be measured by TEM.

Oxide	Size / nm
Bulk $BaTiO_3$	900 ± 250
Ball milled $BaTiO_3$	8.5 ± 3.0
Bulk $PbTiO_3$	260 ± 100
Ball milled $PbTiO_3$	7.4 ± 3.0
Bulk $LiNbO_3$	1800 ± 1100
Ball milled $LiNbO_3$	45 ± 20

Oxide	Size / nm
Bulk $CaTiO_3$	750 ± 370
Ball milled $CaTiO_3$	9.5 ± 3.7
Bulk $SrTiO_3$	920 ± 320
Ball milled $SrTiO_3$	7.7 ± 2.5
Bulk TiO_2	120 ± 40
Ball milled TiO_2	8.0 ± 2.4

Attachment of Rh porphyrin 4b to oxide surfaces. Bulk TiO_2, $CaTiO_3$, $SrTiO_3$, $PbTiO_3$, or $BaTiO_3$ powder (~100 mg) was soaked overnight in a solution of **4b** in CH_2Cl_2 (~1 mM). Subsequently, the powder was allowed to settle overnight and the catalyst solution carefully pipetted off. The powder was then allowed to dry at room temperature and then baked at 150 °C under an atmosphere of N_2. The resulting functionalized bulk powder was rinsed twice with CH_2Cl_2 and sonicated in CH_2Cl_2 (4 × 15 minutes) to remove unattached molecules of **4b**. Particle suspensions were centrifuged (500 rpm) between each wash to facilitate this process. The functionalized powder was stored at room temperature, or subjected to ball milling (see above) before use.

Spontaneous polarization measurements. Spontaneous polarization of nanoparticles was measured through the electric response of cells filled with nanoparticles suspended in a heptane/oleic acid mixture,[2] an insulating non-polar fluid. An electric field applied across the cell aligns the ferroelectric (i.e. polar) nanoparticles such that their dipole moments become parallel to the field. A periodic *ac* electric field causes rotation of the polar nanoparticles, which gives rise to an *ac* displacement current. Integration of the positive (or negative) part of the displacement current density over half a period gives a displacement charge density proportional to the spontaneous polarization of the material, P_s, and the volume fraction of the material in the suspension.[3] The cells were made using approximately 2×3 cm^2 glass plates, coated with a transparent conductive layer of indium tin oxide (ITO) on one side of each plate (the side adjacent to the suspension); a space of 8 microns between the ITO layers was achieved using non-polar glass spacer beads. Examples of the displacement current traces under a triangular voltage waveform are given in **Figure S15** for the ball milled $BaTiO_3$, $CaTiO_3$, $SrTiO_3$, and TiO_2 nanoparticles. For clarity, the background from the cell filled with just heptane/oleic acid mixture (no nanoparticles) was subtracted from the original data.

The data in **Figure S1** show both the result of aggregation as a function of nanoparticle concentration (**Figure S15b**) and the differences between various titanium oxide-based materials (**Figure S15b,c**); **Figure S15a** shows half of the period of the *ac* symmetric triangular voltage

waveform used to measure the displacement currents. With a low enough concentration, where the nanoparticles do not interact with each other,[4] a single feature is observed. With higher concentrations side band structures (wings) form, which are the result of dynamic aggregation and disaggregation of nanoparticles.[4] When comparing the measured displacement current between $BaTiO_3$ (**Figure S15b**) and $CaTiO_3$, $SrTiO_3$, and TiO_2 nanoparticles (**Figure S15c**), one must note the concentrations used in each case. Because of the very weak signal in $CaTiO_3$, $SrTiO_3$, and TiO_2, the concentrations used to get a measurable signal were far greater than the case of $BaTiO_3$. For example, to get a similar displacement current in $CaTiO_3$, 100 times more particles were required with respect to ferroelectric $BaTiO_3$. The signals for $SrTiO_3$ and TiO_2 were noticeably smaller, where the displacement current in TiO_2 was about three orders of magnitude weaker than the measured current for $BaTiO_3$. Despite the relatively large concentration of the particles used to obtain data for **Figure S15c** only a central peak was observed, this lack of aggregation would be expected for suspensions that had limited particle interactions, i.e. little Coulombic interaction,[4] in this case the particles are likely to be predominantly in a paraelectric phase. The spontaneous polarization values of the various titanium oxide-based nanoparticles in suspension were measured with a 5 mHz repetition rate with concentrations that did not result in aggregation. The spontaneous polarization values for each material tested (bulk powders and nanoparticles) are summarized in the table below.

**Material**	**P_s / µC cm^{-2}**
Unmilled $BaTiO_3$	0
Unmilled $BaTiO_3$ annealed under N_2 at 150°C for 24 h	0
Ball milled $BaTiO_3$	20
Unmilled $CaTiO_3$	0
Ball milled $CaTiO_3$	0.19
Unmilled $SrTiO_3$	0
Ball milled $SrTiO_3$	0.05
Unmilled TiO_2	0
Ball milled TiO_2	0.01

Reaction setup. Reactions were performed by diluting freshly ball milled 20:1:1 w/w/w heptane/oleic acid/nanoparticle suspensions to the appropriate nanoparticle concentration with a solution of Rh porphyrin catalyst **4a** in CH_2Cl_2, followed by a solution of **1** in CH_2Cl_2. The reactions were allowed to proceed for 19 h, then analyzed using NMR and high-performance liquid chromatography (HPLC). **2** and **3** were the only major products observed. Results with other solvents besides CH_2Cl_2 are summarized in **Figure 5** in the main text.

NMR analysis. Nuclear magnetic resonance (NMR) data were obtained on a Varian Inova spectrometer at 600 MHz for ^1H nuclei. After the appropriate reaction time had elapsed, reactions or aliquots were quenched with at least 3 volumes of MeCN (Fisher Scientific) and filtered through a 0.22 μm syringe filter to remove flocculated nanoparticles if necessary. The solvent was evaporated and the residue taken up into CD_3CN (Cambridge Isotope Laboratories) to produce a sample for NMR analysis. Relative amounts of **1**, **2** and **3** were obtained through careful integration of the diagnostic peaks (**1**: 5.59 ppm, s, 1H; **2**: 2.31–2.30 ppm, m, 2H; **3**: 6.93–6.90 ppm, dt, 1H) of each substance on the ^1H NMR spectrum (600 MHz, CD_3CN, nt = 200, at = 4 seconds, d1 = 6 seconds) of the solution, from which conversion and product ratio were calculated. The data was found to be consistent with the results from HPLC analysis (see below). Because of the small quantities of products and long acquisition time required per sample, most samples were analyzed only by HPLC. A sample NMR spectrum of the relevant regions for a reaction of **1** with **4a** is shown in **Figure S16**.

HPLC analysis. Reactions were analyzed on an Agilent 1200 HPLC equipped with a Diode Array Detector and an Agilent Eclipse-XDB-C18 reverse phase column. Analysis of **1**, **2** and **3** was carried out using the same method as the literature.[1] If the conversion was less than approximately 5%, two columns in series were used instead with a method of twice the length in order to improve peak separation. Peak areas were calculated after fitting to GaussMod curves in OriginPro 8.5.1, and adjusted for extinction coefficient (see below) to provide conversion and product ratios.

Relative extinction coefficients of 1, 2 and 3. The relative extinction coefficients of **1**, **2** and **3** at 210 nm were determined previously.[1]

Relative extinction coefficients at 210nm:
1: 1.15
2: 1.62
3: 1.00

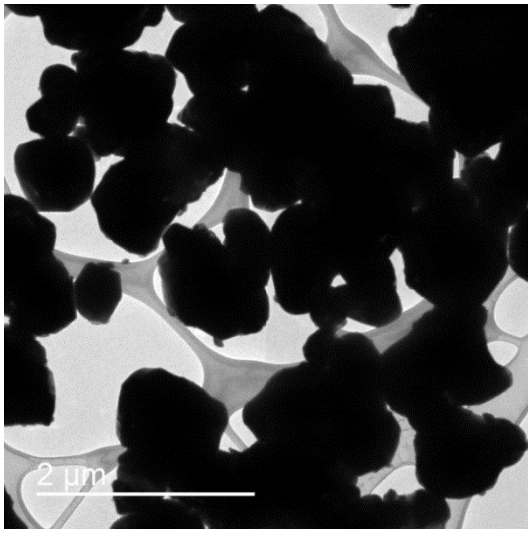

Figure S1. Representative TEM image of bulk $BaTiO_3$ particles.

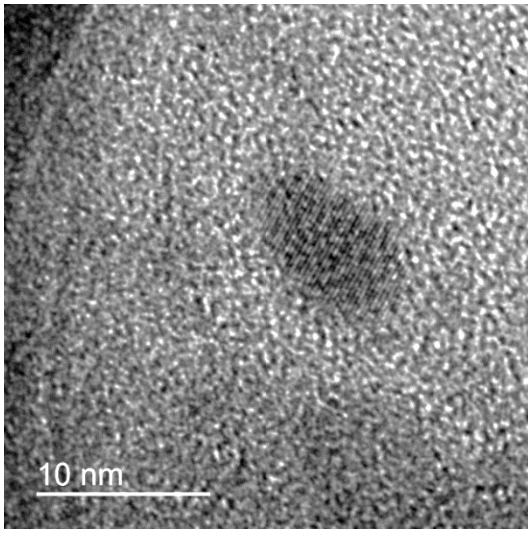

Figure S2. Representative TEM image of ball milled $BaTiO_3$ nanoparticles.

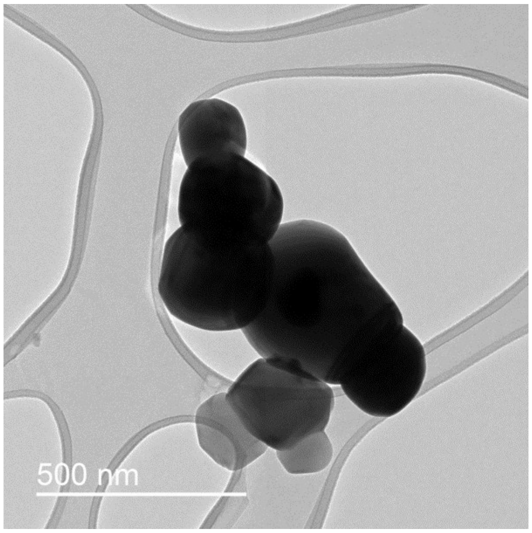

Figure S3. Representative TEM image of bulk $PbTiO_3$ particles.

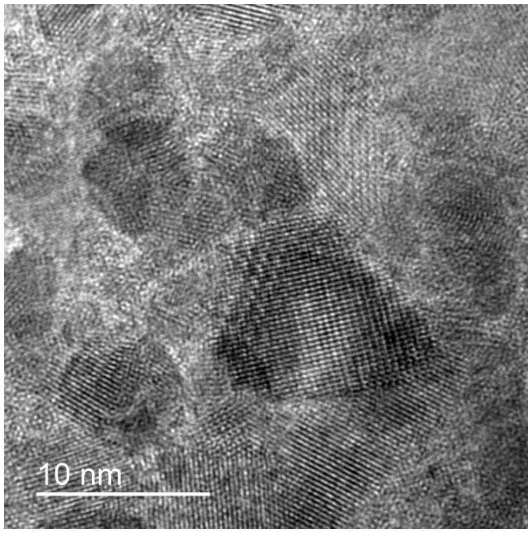

Figure S4. Representative TEM image of ball milled $PbTiO_3$ nanoparticles.

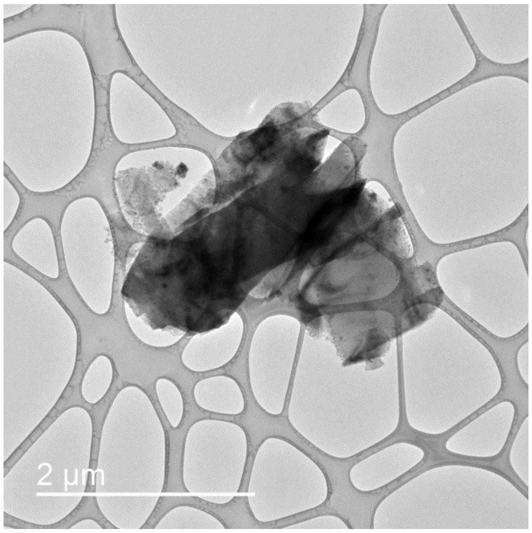

Figure S5. Representative TEM image of bulk $LiNbO_3$ particles.

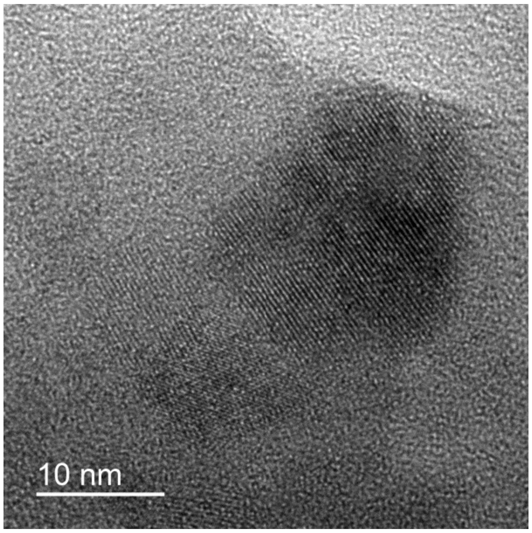

Figure S6. Representative TEM image of ball milled $LiNbO_3$ nanoparticles.

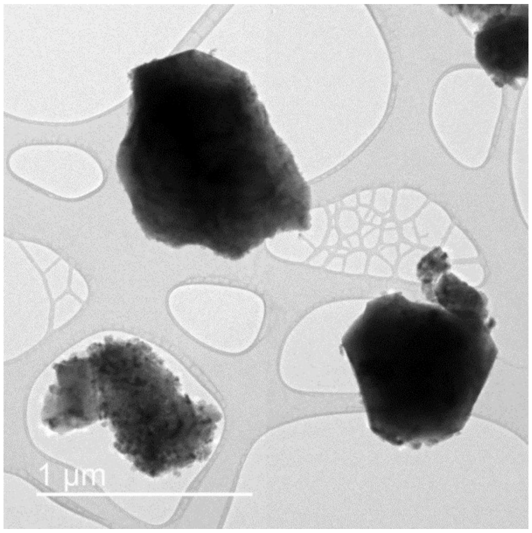

Figure S7. Representative TEM image of bulk $CaTiO_3$ particles.

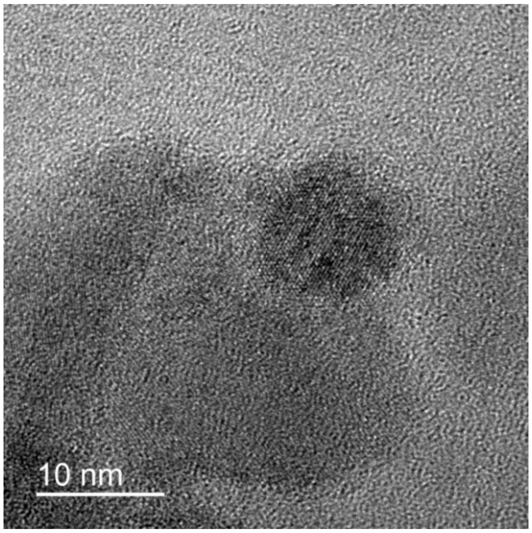

Figure S8. Representative TEM image of ball milled $CaTiO_3$ nanoparticles.

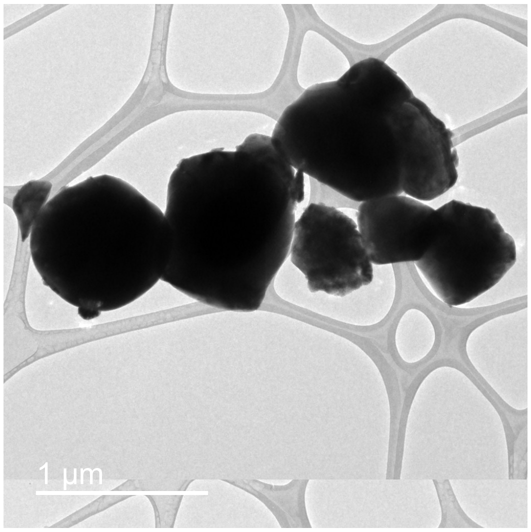

Figure S9. Representative TEM image of bulk $SrTiO_3$ particles.

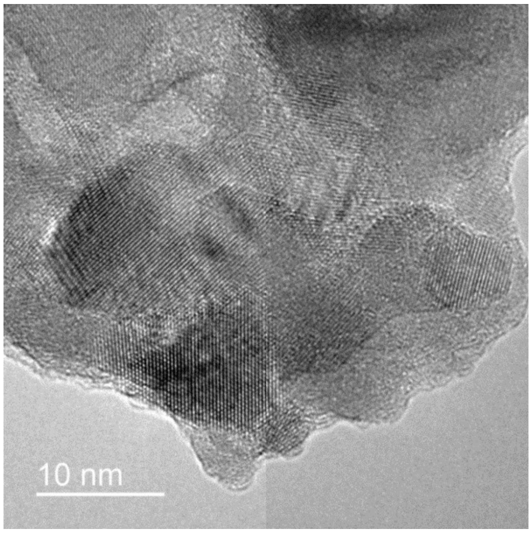

Figure S10. Representative TEM image of ball milled $SrTiO_3$ nanoparticles.

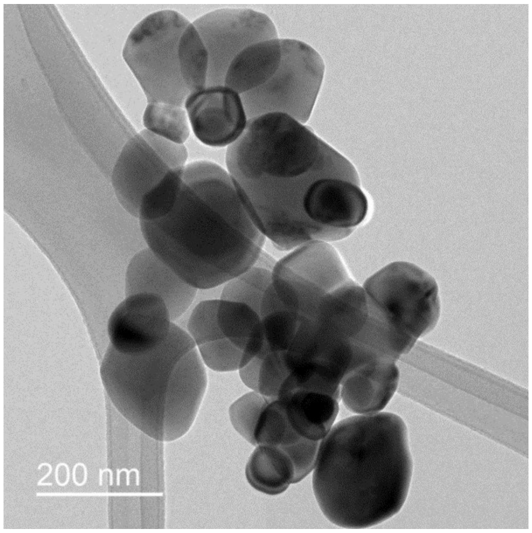

Figure S11. Representative TEM image of bulk TiO_2 particles.

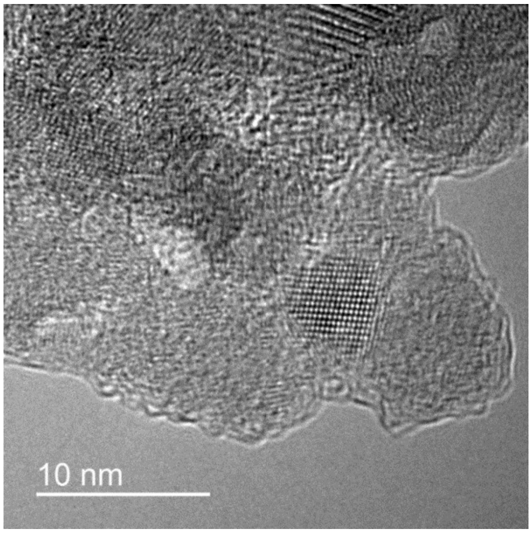

Figure S12. Representative TEM image of ball milled TiO_2 nanoparticles.

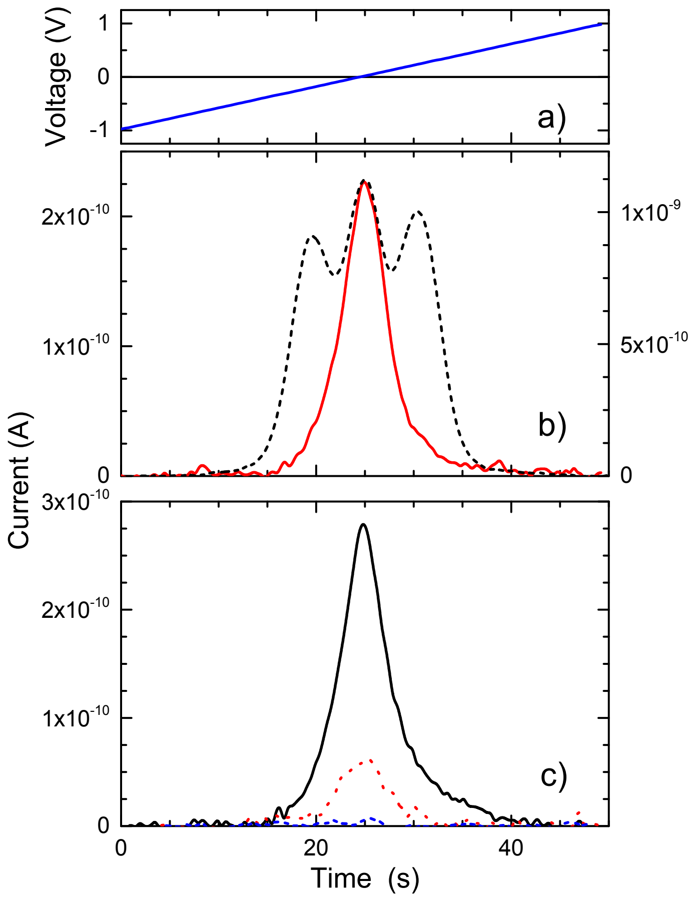

Figure S13. Examples of the displacement current traces under the symmetric triangular voltage waveform across the cells. (a) Half a period of *ac* waveform. (b) Measured displacement current in $BaTiO_3$ nanoparticle suspension, 0.0045 wt.% (solid red line, left y-axis) and 0.045 wt.% (dashed black line, right y-axis). (c) Measured displacement current in 0.45 wt.% $CaTiO_3$ (solid black line), 0.45 wt.% $SrTiO_3$ (dashed red line), and 0.225 wt.% TiO_2 (dotted blue line) nanoparticles.

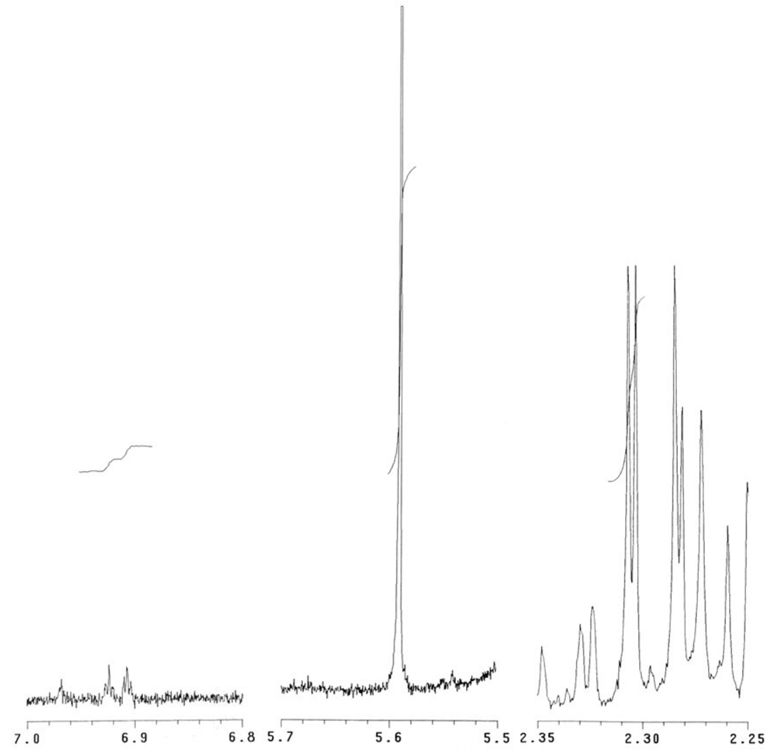

Figure S14. Sample ^{1}H NMR spectrum of a typical reaction mixture quenched in CD_3CN showing the diagnostic peaks for **1** (5.59 ppm, s, 1H), **2** (2.31–2.30 ppm, m, 2H), and **3** (6.93–6.90 ppm, dt, 1H). In this particular experiment, the ratio of **2**:**3** is determined to be 3.8:1.0 with 27.4% conversion. Oleic acid, which is present whenever $BaTiO_3$ nanoparticles are used, contributes a large number of other peaks elsewhere in the spectrum.

Experimental data for Figure 2. The observed ratios of **2**:**3** with various concentrations of **1**, catalyst **4a** and ball milled $BaTiO_3$ (or other oxide) in CH_2Cl_2 are summarized below. This data is presented in **Figure 2** in the main text.

**[1] / mM**	**[4a] / µM**	**[$BaTiO_3$] / mg mL^{-1}**	**[oxide] / mg mL^{-1}**	**2:3**	**% Conversion**
2	20	0	0	11.5 ± 0.1	98.3 ± 1.0
2	20	0.01	0	12.0 ± 0.3	97.4 ± 0.3
2	20	0.1	0	11.8 ± 0.5	97.5 ± 0.2
2	20	1	0	10.0 ± 1.6	97.2 ± 0.2
2	2	0	0	11.0 ± 0.2	94.3 ± 0.3
2	2	0.01	0	7.9 ± 0.1	61.9 ± 3.1
2	2	0.1	0	7.1 ± 0.1	54.3 ± 0.6
2	2	1	0	6.3 ± 0.2	45.2 ± 0.6
0.2	0.2	0	0	10.7 ± 0.3	45.3 ± 1.8
0.2	0.2	0.01	0	4.8 ± 0.8	41.6 ± 0.5
0.2	0.2	0.1	0	3.7 ± 1.0	32.1 ± 4.7
0.2	0.2	1	0	2.1 ± 0.4	21.3 ± 0.7
0.2	0.2	0	1 ($PbTiO_3$)	2.1 ± 0.1	8.4 ± 0.3
0.2	0.2	0	1 ($LiNbO_3$)	4.5 ± 0.4	15.4 ± 1.4
0.2	0.2	0	1 ($CaTiO_3$)	11.1 ± 1.3	13.5 ± 0.1
0.2	0.2	0	1 ($SrTiO_3$)	11.0 ± 1.5	13.3 ± 0.6
0.2	0.2	0	1 (TiO_2)	9.5 ± 0.3	80.2 ± 2.9

Experimental data for Figure 3. The observed ratios of **2**:**3** with 2 mM of **1** and 2 μM **4a** in CH_2Cl_2 at room temperature after various reaction times are summarized below. Ball milled oxide, if present, is used at a concentration of 1 mg mL^{-1}. This data is presented in **Figure 3** in the main text.

Reaction Time / h	Ball Milled Oxide	2:3	% Conversion
6	No oxide	9.8 ± 0.3	50.4 ± 0.9
18	No oxide	10.0 ± 0.5	66.3 ± 0.3
30	No oxide	9.9 ± 0.1	70.7 ± 0.2
48	No oxide	9.8 ± 0.4	73.4 ± 1.1
72	No oxide	9.9 ± 0.3	75.5 ± 0.6
96	No oxide	9.9 ± 0.3	76.9 ± 0.4
6	$BaTiO_3$	4.6 ± 0.3	8.7 ± 0.4
18	$BaTiO_3$	5.7 ± 0.4	13.9 ± 1.0
30	$BaTiO_3$	6.1 ± 0.5	17.3 ± 1.5
48	$BaTiO_3$	6.1 ± 0.3	20.2 ± 1.1
72	$BaTiO_3$	5.9 ± 0.3	23.1 ± 1.2
96	$BaTiO_3$	5.7 ± 0.1	26.3 ± 1.6
6	$PbTiO_3$	6.1 ± 0.4	8.2 ± 0.3
18	$PbTiO_3$	6.3 ± 0.2	12.3 ± 0.5
30	$PbTiO_3$	7.6 ± 0.6	15.1 ± 0.3
48	$PbTiO_3$	7.0 ± 0.2	20.0 ± 0.9
72	$PbTiO_3$	6.4 ± 0.3	21.3 ± 0.8
96	$PbTiO_3$	7.2 ± 0.2	25.9 ± 1.3
6	$LiNbO_3$	5.5 ± 0.3	14.0 ± 0.6
18	$LiNbO_3$	6.1 ± 0.5	19.5 ± 0.2

Experimental data for **Figure 3**. (continued)

Reaction Time / h	Ball Milled Oxide	2:3	% Conversion
30	$LiNbO_3$	6.6 ± 0.1	25.3 ± 1.0
48	$LiNbO_3$	6.7 ± 0.2	30.4 ± 1.0
72	$LiNbO_3$	6.7 ± 0.1	34.2 ± 0.7
96	$LiNbO_3$	6.3 ± 0.1	39.9 ± 0.1

Experimental data for Figure 4. The effect of adding ball milled $BaTiO_3$ on the observed ratios of **2**:**3** with 2 mM of **1** and 2 μM of catalyst **4a** in various solvents is summarized below. This data is presented in **Figure 4** in the main text.

[1] / mM	[4a] / μM	[$BaTiO_3$] / mg mL^{-1}	Solvent	2:3	% Conversion
2	2	0	$PhCF_3$	17.5 ± 0.9	71.5 ± 25.6
2	2	1	$PhCF_3$	8.8 ± 0.3	33.6 ± 4.0
2	2	0	CH_2Cl_2	11.0 ± 0.2	94.3 ± 0.3
2	2	1	CH_2Cl_2	6.3 ± 0.2	45.2 ± 0.6
2	2	0	THF	17.9 ± 0.5	32.8 ± 23.7
2	2	1	THF	2.7 ± 0.7	7.7 ± 0.7
2	2	0	EtOAc	9.4 ±1.5	88.3 ± 11.1
2	2	1	EtOAc	7.2 ± 0.4	27.2 ± 6.8
2	2	0	PhMe	15.3 ± 0.7	98.0 ± 1.2
2	2	1	PhMe	10.2 ± 0.9	64.0 ± 11.7

Experimental data for Figure 5. The observed ratios of **2**:**3** arising from the reaction of **1** with catalyst **4b** unattached or attached to TiO_2 or various titanates in CH_2Cl_2 under various conditions are summarized below. This data is presented in **Figure 5** in the main text.

[1] / mM	[4b] / µM	[$BaTiO_3$] / mg mL^{-1}	$BaTiO_3$ treatment	Reaction Time / h	2:3	% Conversion
2	2	0	—	19	8.8 ± 0.6	89.3 ± 8.2
2	2	1	Ball milled, unfunctionalized	19	4.5 ± 1.3	30.8 ± 3.1
2	Attached to TiO_2	1	Unmilled after functionalization	19	6.3 ± 0.1	2.9 ± 0.1
2	Attached to $CaTiO_3$	1	Unmilled after functionalization	96	6.2 ± 0.1	8.2 ± 0.4
2	Attached to $SrTiO_3$	1	Unmilled after functionalization	96	6.2 ± 0.3	3.1 ± 0.6
2	Attached to $PbTiO_3$	1	Unmilled after functionalization	96	5.3 ± 0.1	2.1 ± 0.1
2	Attached to $BaTiO_3$	1	Unmilled after functionalization	19	2.2 ± 0.1	9.5 ± 0.5
2	Attached to $BaTiO_3$	1	Ball milled after functionalization	19	1.8 ± 0.7	5.1 ± 2.2

References

1. Gorin, C. F.; Beh, E. S.; Bui, Q. M.; Dick, G. R.; Kanan, M. W. *J. Am. Chem. Soc.* **2013**, *135*, 11257.
2. Atkuri, H.; Cook, G.; Evans, D. R.; Cheon, C. I.; Glushchenko, A.; Reshetnyak, V.; Reznikov, Y.; West, J.; Zhang, K. *J. Opt. A – Pure Appl. Opt.* **2009**, *11*, 024006.
3. Basun, S. A.; Cook, G.; Reshetnyak, V. Y.; Glushchenko, A. V.; Evans, D. R. *Phys. Rev. B* **2011**, *84*, 024105.
4. Evans, D. R.; Basun, S. A.; Cook, G.; Pinkevych, I. P.; Reshetnyak, V. Y. *Phys. Rev. B* **2011**, *84*, 174111.
